# A Comprehensive Review of Biodegradable Polymer-Based Films and Coatings and Their Food Packaging Applications

**DOI:** 10.3390/ma15175899

**Published:** 2022-08-26

**Authors:** Vatsla Gupta, Deblina Biswas, Swarup Roy

**Affiliations:** School of Bioengineering and Food Technology, Shoolini University, Bajhol, Solan 173229, India

**Keywords:** biopolymers, edible packaging, films and coatings, active packaging

## Abstract

Food sectors are facing issues as a result of food scarcity, which is exacerbated by rising populations and demand for food. Food is ordinarily wrapped and packaged using petroleum-based plastics such as polyethylene, polyvinyl chloride, and others. However, the excessive use of these polymers has environmental and health risks. As a result, much research is currently focused on the use of bio-based materials for food packaging. Biodegradable polymers that are compatible with food products are used to make edible packaging materials. These can be ingested with food and provide consumers with additional health benefits. Recent research has shifted its focus to multilayer coatings and films-based food packaging, which can provide a material with additional distinct features. The aim of this review article is to investigate the properties and applications of several bio-based polymers in food packaging. The several types of edible film and coating production technologies are also covered separately. Furthermore, the use of edible films and coatings in the food industry has been examined, and their advantages over traditional materials are also discussed.

## 1. Introduction

Films and coatings are types of packaging materials used widely in the food industry for a variety of purposes. For example, they serve as protection against physical, chemical, as well as biological damage to food. They are also helpful in preventing the loss of aroma, flavor, antimicrobials, and antioxidants [1]. They prevent oxygen, carbon dioxide, and moisture loss, and are hence helpful in increasing the shelf life of food and improving the mechanical integrity and handling characteristics of food [2]. The production and usage of petroleum-based polymer-generated plastic for manufacturing coatings and films have increased over recent decades. According to Derraik et al. (2002), plastic can be defined as a synthetic or semisynthetic organic polymer having good barrier properties against moisture, oil, and gases, with strength characteristics and good resistant properties against stress and corrosion [3]. It is cheap, lightweight, and easy to carry and handle, making it highly convenient for daily use. Due to its abundant usage in various applications, the plastic industry in India is worth between 3000 and 4000 crores [4]. It is believed that half of all plastic products which are used in the food industry such as cutlery, plastic bags, coatings, and packaging material, are designed to be thrown away. Moreover, the world production of plastics grew from 1.5 million metric tons in 1950 to 359 million metric tons in 2018 [5]. According to Sabbah et al. (2017), more than 35 million tons of waste derived from various plastic products are produced each year in the world and unfortunately only 7% of these are recycled [6].

Moreover, due to the presence to toxic chemicals which are used in the manufacturing of plastic, its usage and disposal has resulted in hazardous effects related to environmental as well as human health [7]. The current scenario of plastic pollution indicates that approximately 400 thousand plastic bottles and about 700 thousand plastic bags are disposed of worldwide every minute [6]. As polymer is non-biodegradable by nature, it remains in the environment, leading to severe environmental as well as health hazards. Most plastics and other petrochemical polymers are considered “hard-to-degrade” because of their corrosion-resistant qualities, and they can remain in the environment for decades or even centuries [8]. One of the serious issues which can arise due to this is biomagnification, caused when a certain amount of toxic material (after leaching from the polymer into soil/bodies of water) reaches the food chain and its concentration level increases after each successive level in the food chain, creating a health hazard for various organisms. Other hazards include soil pollution, water pollution, air pollution, groundwater contamination, etc.

High-density polyethylene (HDPE), low-density polyethylene (LDPE), polyvinyl chloride (PVC), polystyrene (PS), polypropylene (PP), and polyethylene terephthalate (PET) are some of the most widely used and plentiful petrochemical-based polymers (Table 1), accounting for almost 90% of the total plastic output worldwide [7].

A study conducted by Patil et al. in (2018) demonstrated that India generated almost 15,000 tons of plastic waste every day [10]. This amount of plastic waste generation is a result of an increasing population, which reached 7.7 billion in 2019 and is expected to reach around 9.7 billion in 2050, as per UN reports published in 2019 [11]. As there is a high demand for packaged food, the demand for packaging material is also high [12]. This demand has led researchers to develop an appropriate packaging and coating material which can increase the shelf life of food, contribute to sustainable development through the utilization of waste, and also minimize the pollution caused by single-use plastic packaging materials.

As a result, the world is now focusing on bio-based edible films and coatings that can easily degrade after use, thereby also contributing to waste reduction. Biodegradable and edible films and coatings are an integral aspect of food packaging; they are formed from hydrocolloids such as proteins, polysaccharides, lipids, antimicrobial components, or a combination of these. They consist of a continuous thin layer (between 0.050 and 0.250 mm) which is safe for consumption along with food [13,14]. Films are mainly used to wrap food material, whereas coatings can be used directly on food products [15]. The types of materials used for the production of these films are mostly renewable biological sources, mainly consisting of starch, cellulose, hemicellulose, protein, gelatin, lipids, fibers, etc. Examples of such materials include corn, sorghum, rice, fruit and vegetable waste, and agricultural waste such as wood chips or bagasse. Waste produced by food industries such as cane molasses from the sugar industry, barley spent grain from breweries, etc., could be a good source of these bio-based films as they are produced throughout the processing of food from agricultural land to food processing units through to the consumer. They are easily available at a low cost and their usage would help in the waste utilization process [16]. However, unlike petroleum-based polymers, these materials do not offer good barrier and mechanical properties; therefore, some additives are also required to enhance these properties. A single layer of edible coating may not be sufficient to withstand all the barrier requirements; therefore, a multilayer coating consisting of a combination of more than two types of bio-based coating materials can improve its overall characteristics. In this context, nanotechnology has also been introduced to improve the mechanical strength and barrier properties of edible packaging materials [17]. Bio-based edible films and coatings are appropriate for packaging fruits, vegetables, dairy, and meat-based products at a commercial level. Although bio-based polymers are promising, the production cost is a key limitation for its practical use. One of the strategies to reduce costs is the mass production and increased awareness of biopolymer-based packaging. In addition, extra attention is required for water-insoluble bio-based polymers in order for them to be properly disposed of. Nowadays, research is mainly focused on the use of biodegradable edible coatings for fresh and minimally processed fruits and vegetables such as avocados [18], strawberries [19], pears [20], mangos [21], etc. The type of edible packaging material selected is dependent upon the type of food, for example, fruit, dairy, or meat, and the storage conditions, including temperature and relative humidity [22]. Because different foods have different qualities, each property must be safeguarded, diminished, or enhanced in order to maintain product safety. The manner in which the coating is applied is also very important both in terms of cost and efficacy [23].

The application of edible coatings and films on food products is not novel; it has been applied onto food products for the preservation of food for centuries. For instance, wax- and cellulose-based coatings on highly perishable foods such as meat, fruits, and vegetables have been applied since the 12th century in China [24,25]. Edible films and coatings act as a semi-permeable barrier, preventing microbes from entering the meal without changing the original contents. They increase the shelf life of food by lowering the moisture and reducing the dissolved component migration, gas exchange, oxidative reactions, and respiration rates [25,26]. However, they must give the coated product a suitable color, fragrance, taste, flavor, and texture [27,28]. The efficiency of edible films or coatings is determined by three aspects: (i) the biomaterials used, (ii) the strategic and functional parameters of their application to food products, and (iii) the food product’s specific requirements [23].

The current study’s goal is to examine a specific selection of conventional materials based on their qualities, their application on foods, and the special requirements of food products, as well as various preparation methods. We also give an overview of the current research work on biopolymers and their implications for food packaging.

## 2. Sources of Biodegradable Material

**Figure 1:**
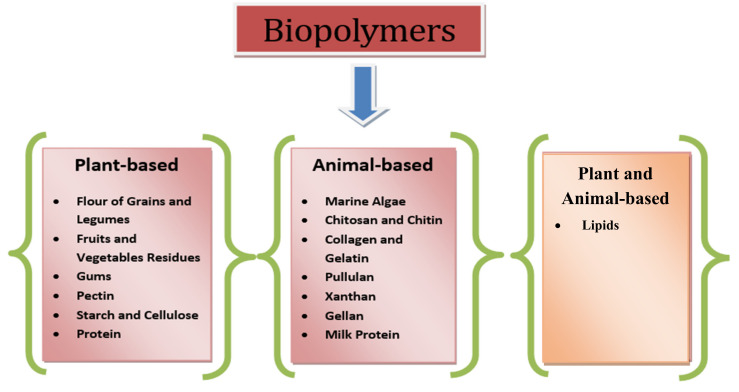
Different sources of biomaterials.

A vast range of packaging materials obtained from inexhaustible resources has been developed in the last few years. Many plant and animal by-products are high in polysaccharides and proteins, which can be used to make edible or biodegradable coatings and film compositions. Commercial opportunities exist for alternative and novel materials made from underutilized food items, renewable resources, and the valorization of agro-industrial and marine waste. In a circular economy, using these resources adds value to waste products and provides an enticing alternative to nonrenewable sources. Furthermore, the vast majority are not only biodegradable, but also edible. These by-products could come from underutilized foods, renewable resources, and the valorization of agro-industrial and marine wastes, among other sources. Husks, feathers, shells, skins, seeds, peels, stems, and leaves are examples of by-products with significant value potential [29]. As the natures of these polymers are hydrophilic, i.e., they attract water easily, they therefore exhibit poor water vapor barriers and mechanical properties in comparison to plastic materials. That is why current research is mainly focused on improving these properties of polymers by incorporating reinforcing agents such as nano particles, bioactive functional compounds, etc., to make them more water resistant, similarly to plastic materials, in order to meet the functionality requirements of food packaging materials [30]. The various types of bio-based polymers commonly used in food packaging are schematically tabulated in Figure 1.

### 2.1. Plant-Based Material

#### 2.1.1. Flour of Grains and Legumes

**Figure 2:**
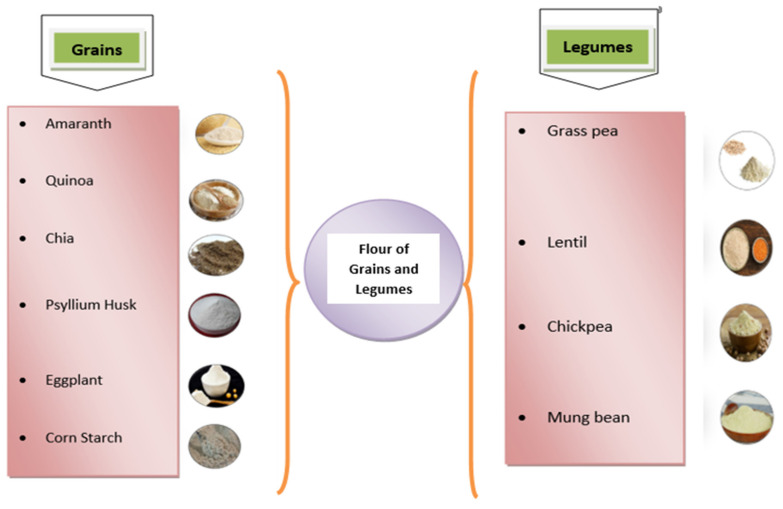
Grain and legume flours used to produce films and coatings [13].

There has been a lot of research into edible films and coatings based on polysaccharides or proteins from traditional crops and roots [24]. Figure 2 lists the various materials that can be used to produce films.

According to Tóth and Halász (2019), psyllium husk and its flour are excellent sources for the making of packaging film [31]. When comparing the mechanical and water vapor properties of edible film made from eggplant flour and corn starch to film made without corn starch, Nouraddini et al. (2018) concluded that the mechanical and water vapor qualities of the former were compromised [32]. Components such as lipids, fibers and proteins exist in the edible polymers, and this could be the reason for these compromised physical properties. It has been reported that after 14 days these films entirely decayed, which demonstrates their excellent biodegradability [13].

Legumes have high nutritional value, consisting of high amounts of starch proteins, lipids, vitamins, and minerals. Due to this, they could be an excellent source for film formulations. The research work of Montalvo-Paquini et al. (2018) was based on popular Mexican beans including alubia, flor de mayo, garbancillo, peruano, pinto, mantequilla and negro, and an extracted bean protein concentrate was utilized as a protein source for edible films [33]. Navy beans are one of the largest pulse crops in the USA. It is pea-sized white legume and contains a high total amylose content, which may increase the elastic modulus of films and hence help to decreasing the water vapor permeability. Therefore, Zhang and Li (2021) prepared a film from navy bean starch and concluded that it has better mechanical and water barrier properties when compared to corn starch [34]. Ochoa-Yepes et al. (2018) developed composite films from cassava starch films with the addition of a residue of lentil protein production [35]. These films were more water resistant and mechanically stable, and were fully degradable. Moreover, they were able to tolerate temperatures of up to 240 °C. Kocakulak et al. (2019) worked on the production of edible films made from chickpeas with the addition of gallic acid, which was tested using different pH levels (9 and 11). Their findings concluded that at pH 11, the films have higher antioxidant activity and have better mechanical strength as compared to composite films; they can therefore be used for wrapping foods which are prone to oxidation [36].

#### 2.1.2. Fruits and Vegetables Residues

The food sector generates considerable amounts of solid residue each year. Furthermore, these residues are high in nutrients and bioactive substances, as well as biopolymers such polysaccharides and dietary fibers [37]. Biopolymers derived from food waste are known for their ability to create films, which is the subject of the current research. Fruit and vegetable purees have been widely used as components of hydrocolloid films and coatings in prior studies [38]. Fruit and vegetable wastes, which are typically processed into flour, show promise as they contain film-forming components [39]. Many research articles have been reported recently regarding the use of fruits and vegetables as ingredients in edible films and coatings [40,41,42,43]. New edible films, based on flour including orange, passion fruit, watermelon, lettuce, courgette, carrot, spinach, mint, taro, cucumber, and rocket residue, were investigated [39]. Notably, without the use of plasticizers, the scientists were able to produce uniform, flexible films with promising properties. Furthermore, the addition of flour created from potato skin residue increased mechanical resistance [13]. Erica Ayquipa-Cuellar et al. (2021) obtained edible films from prickly pear peel mucilage and potato husk starch [44]. The film showed good barrier properties and physico-chemical properties due to the presence of glycerol, which is used as plasticizer. Therefore, these films can be used to wrap fruits and vegetables. Plant residues are complex materials in general, and so depending on their composition and particle size, distinct residue fractions could have varied applications, such as for the use in dietary fiber or as primary components for edible films and coatings [13].

#### 2.1.3. Gums

Gum arabic, gum karaya, gum ghatti, mesquite, and tragacanth gum are just a few of the natural, plant-derived gum exudates that have been found in recent decades. Gum Arabic (or acacia gum) is a polysaccharide obtained from the gummy exudate of the stems and branches of the species of the genus Acacia, most often of the species *Acacia senegal* (L.) Wild. var. senegal. Gum arabic can also be obtained from the following species: *Acacia senegal*, *Acacia seyal*, *Acacia polyacantha*, *Acacia gerrardii*, and *Acacia laeta* [45]; it is one of the oldest and most well-known natural gums, having been used for over 5000 years. Gum arabic has a wide range of applications in the food, paint, and textile industries because to its emulsifying, stabilizing, thickening, and binding properties [46]. Furthermore, gum Arabic has the potential to be used as a protective edible covering for the extension of the shelf life of food goods, such as pecan nuts, by eliminating their moist and oily appearance [47].

Almond gum, also known as Persian gum, is a new gum exudate obtained from the almond tree’s trunk and branches. Bashir and Haripriya (2016) compared gum arabic and almond gum and found that almond gum has better physical features than gum arabic, such as bulk density, oil holding capacity, flowability, and mineral content [48].

Guar gum is a biopolymer which is hydrophilic in nature and is made from the seeds of the *Cyamopsis tetragonoloba* plant. Because of the huge number of hydroxyl groups, guar gum can form a homogenous edible film that is nearly water soluble [49,50].

Xanthan gum is an extracellular heteropolysaccharide made from a pure Xanthomonas campestris culture by submerged aerobic fermentation [51,52]. It is employed as an additive to starch-based films, resulting in an increase in some of their mechanical qualities, due to its capacity to form very viscous solutions at low concentrations and its biodegradability [53].

Polysaccharide-gum-based edible films and coatings create a semi-permeable barrier. Although weight loss may occur and the rate of respiration at the surface may be slowed, this helps to maintain the nutritious value of fruits and vegetables. This form of edible coating not only extends the shelf life of fruits and vegetables and prevents quality deterioration during storage, but also protects them from pathogens [54,55].

Robles-Flores et al. (2018) produced edible coatings from *Cajanus cajan* seed protein isolate and gum, which were successfully employed in improving the quality of coated strawberries [56]. Pinho tree seed flour and starch are good sources of complex carbohydrates with outstanding film-forming characteristics [57].

#### 2.1.4. Pectin

Pectin is the fibrous component extracted from plant cell walls. It is a complex anionic polysaccharide composed of β-1,4-linked α-d-galacturonic acid residues, where the uronic acid carboxyl is either fully (high methoxy pectin) or partially (low methoxy pectin) methyl-esterified [58,59,60]. Research findings suggest that films produced from pectin have excellent mechanical properties as the raw material of pectin can act as a natural plasticizer, which helps in improving the extensibility of films [61,62]. Because pectin-based films are poor moisture barriers, they are only suggested for low-moisture foods [54,63]. Recent studies on production and the use of active films based on pectin (HMP) or pectin-containing plant products (e.g., fruit, vegetable, and hibiscus purees) with several natural antimicrobials (e.g., carvacrol and cinnamaldehyde, the main ingredients of oregano and cinnamon oil, respectively) have revealed the efficacy of these materials (dose-dependent bactericidal activity) to reduce the growth of foodborne pathogens (e.g., *E. coli* O157:H7, *S. enterica*, *Campylobacter jejuni* and *Listeria monocytogenes*) on meat and poultry products [64,65,66]. Therefore, pectin-based films with some additives such as natural herbs can make them effective for high moisture food as well. Research findings by Sucheta et al. (2019) suggested that composite films based on a starch and pectin in ratio of 1:1 or 60:40 can improve the mechanical, thermal, and structural properties of edible films [67].

#### 2.1.5. Starch and Cellulose

Starch is a low-cost carbohydrate-based polymer that is obtained from a wide range of crops. The granules of native starch have a granular form and are made up of a combination of amylopectin (75%) and amylose (25%). d-glucopyranosyl units in the linear molecule amylose are connected by a-1,4 bonds. Amylopectin is a very large, highly branched molecule that contains a-d-glucopyranosyl units that are connected by a-1,4 and a-1,6 bonds [68]. Starch is mainly found in plants in tubers, seeds, and roots. The other sources of starch which are of industrial use are obtained from maize, wheat, edible cassava, potato, amaranth, and quinoa [68,69]. Their membrane- and gel-forming properties are due to their linear structure, which forms hydrogen bonds between hydroxyl groups of two parallel oriented chains, which tends to reduce the affinity of polymer to water [70]. Edible films with higher amylose content have better film-forming properties, i.e., better mechanical strength, elongation, and gas barrier properties. Starch-based films can be a good source for edible films and coatings due to good barrier properties against gases, cohesive strength, and durability. Additionally, these are tasteless, odorless, colorless, and nontoxic in nature. The mechanical properties of these films are, however, compromised due to high water vapor permeability [24].

Cellulose is most abundant natural polymer comprising d-glucose units linked through β-1,4-glycosidic bonds. Due to the large number of intramolecular hydrogen bonds, cellulose is insoluble in water; therefore, it cannot be directly used for film formation. Cellulose is treated with an alkali to swell its cell wall so that it can become soluble in water. For this, a reaction with methyl chloride, chloroacetic acid, or propylene oxide is carried out to obtain methyl cellulose (MC), carboxymethyl cellulose (CMC), hydroxypropyl cellulose (HPC) or hydroxypropyl methyl cellulose (HPMC) [71]. According to Krochta et al. (1997) the characteristics of cellulose, including its gas and moisture barrier, were directly proportional to its molecular weight, and the higher the molecular weight, the better the properties [72]. Edible films made of CMC provide an effective barrier against oxygen and carbon dioxide, but they have poor water barrier properties [73,74]. Mixing carboxymethyl cellulose with other polymers, such as starch and chitosan, has also been discovered to exacerbate the inherent limitations of CMC [54].

#### 2.1.6. Proteins

Proteins typically occur as either fibrous proteins or globular proteins. Because of the chain-to-chain contact, protein-based films or coatings are regarded as highly efficient oxygen blockers, even at low relative humidity (RH). Protein-based films are hydrophilic, which means they have low to moderate water barriers and lose their film-forming properties when exposed to a highly humid environment. They provide a good barrier against hydrophobic chemicals such as oil and aroma. Antimicrobial and antioxidant substances are also present in protein-based edible films [75]. Various globular proteins, such as soy protein, wheat gluten, whey protein, and maize zein, have been investigated by researchers for their ability to form films or coatings [24]. Zein from corn and soy has demonstrated good film- and coating-forming characteristics. Zein is a corn-extracted hydrophobic protein. It can be utilized as an active edible packaging material to preserve the quality and shelf life of food goods due to its inherent antibacterial and antioxidant capabilities [76]. Films and coatings from zein protein are formed by first drying the aqueous ethanol solution of zein followed by the addition of plasticizer to improve flexibility. These films are proven to have good moisture barrier properties [24,77]. Soy protein is another kind of plant protein which is obtained from the soyabean. It has been reported that boiling soy milk to remove the water content results in a soy protein film being formed [24]. The air-drying process also follows this step. Soy protein films have high gas barrier properties compared to lipid- and polysaccharide-based films. Wheat protein (gluten) also has film-forming properties due to its cohesiveness and elastic behavior. The purity of gluten affects the appearance and mechanical properties of films. The addition of plasticizers such as glycerol or sorbitol enhances the flexibility of films and coatings [76].

### 2.2. Animal-Based Material

#### 2.2.1. Marine Algae

Alginate, carrageenan, and agar are polysaccharide-based products extracted from marine algae. Alginate, also known as sodium salt of alginic acid, is obtained from brown seaweed, and possesses the ability to form films [13,78]. Divalent cations such as Ca, Mg, Mn, Al, and Fe are utilized to make alginate coating materials, which are employed as gelling agents [79,80]. Alginate exhibits excellent film-forming properties, imparting a uniform, transparent, glossy look to films. Alginate films are impervious to oils and fats and have high water vapor permeability similar to other hydrophilic polysaccharide [71]. Compared to other biopolymers, alginate has an idiosyncratic colloidal property, which contains a stabilizing, thickening, suspending film or coating, producing a gel-forming and stabilizing emulsion [80,81]. Alginate provides a number of advantages, including reduced shrinkage, and the retention of moisture, food color, and odor [79]. Due to its moisture-retaining properties, it can be used to protect fruits and vegetables from the loss of turgor as moisture is lost from protected part of edible plant only after moisture is lost from the coating. Due to the good barrier properties against gases, alginate films are helpful in delaying lipid oxidation and senescence, and in reducing weight loss in fruits and vegetables [71].

Carrageenan is a natural hydrophilic polymer with a linear chain of partially sulfated galactans that have high membrane formation potential [82,83]; they are most commonly used as coatings [84,85]. Edible films based on iota carrageenan have good mechanical properties as they are emulsion stabilizers and can reduce oxygen transfer and limit surface dehydration and the deterioration of fruit flavor [86]; due to this, they can effectively protect vegetables and fruit against the loss of moisture and turgor, the oxidation of compounds and ageing processes, and can reduce the number of microorganism if combined with ascorbic acid [78].

Agar, extracted from red seaweed, is basically a combined form of agarose (gelling fraction) and agaropectin (non-gelling fraction) [87,88]. Agarose forms a supporting structure in the cell walls of red algae and is responsible for the gelling capabilities of agar, which makes it suited to the production of edible coatings on vegetables and fruit [89]. Agar is noted for its hydrophilic nature and ability to generate robust, thermo-reversible gels. Under normal conditions, agar-based films and coatings are clear, robust, and inflexible, and they are activated with water [90].

#### 2.2.2. Chitosan and Chitin

Chitin is a biopolymer found in the exoskeletons of crustaceans (shrimps, oysters, krill, crabs, squid, and lobsters), the cell walls of filamentous fungi (Mucoraceae), and in other biological materials such as the exoskeletons of arachnids and insects (bumblebees, crickets, bees, silkworm larval skin) [91,92,93].

Chitosan is obtained by N-deacetylation of chitin in an alkaline environment [94]. It is a copolymer made up of β-(1-4)-2-acetamido-d-glucose and β-(1-4)-2-amino-d-glucose units, with the latter accounting for more than 60% of the total [95]. Chitosan is a nontoxic polymer consisting of antifungal, anti-allergenic, antimicrobial, and anti-tumor properties. Due to its properties such as selective gas permeability (only for CO_2_ and O_2_), good mechanical properties, biocompatibility, and biodegradability it is environmentally friendly and is considered a good film-forming material [96,97,98,99,100,101]. Due to its ability to regulate gas permeability, edible chitosan coatings are applied to the surface of fruits and vegetables to reduce their respiration rate [102]. However, its limitations include low water solubility, due to which it forms a rigid crystal structure; additionally, its high water vapor permeability is not useful for humid environments [95,98,103].

#### 2.2.3. Collagen and Gelatin

Collagen, the most ubiquitous protein in the body, can be used for a variety of purposes [104,105]. Collagen is frequently combined with other biopolymers in packaging applications. The agar–alginate–collagen film with silver nanoparticles, for example, has outstanding antibacterial capabilities (against *Listeria monocytogenes* and *Escherichia coli*) as well as strong mechanical and water resistance [106]. According to a study conducted in 2017, collagen was crosslinked with keratin (a protein taken from wool, bird feathers, skin, or hair), which improved the thermal resistance and mechanical capabilities of the collagen-based film [107].

Gelatin is a hydrophilic protein-based polymer derived from the collagen present in animal skin (such as fish, pork, bovine) and bones [108,109,110]. Bovine gelatin films have been found to have a hydrophobic surface and adding chitin to the gelatin film boosts the hydrophobicity even more [111]. Anthocyanins derived from red cabbage were introduced into a fish gelatin film, which demonstrated not only antioxidant action, but also improved the mechanical and water resistance properties of the film [112].

#### 2.2.4. Pullulan

Pullulan is an abundant homopolysaccharide consisting of maltotriose units connected to each other by an α-(1,6)-glycosidic bond. It is synthesized by the yeast-like fungus Aureo basidium pullulans and has various advantages over other polysaccharides in terms of generating edible films [113]. Pullulan films are tasteless, colorless, heat and oil-resistant, have low oxygen permeability, and can be combined with other biopolymers and plasticizers to modify their mechanical and gas barrier properties in a controlled way [114,115,116]. Pullulan’s efficacy as an edible covering for strawberries is comparable to that of alginate, which is commonly utilized in the field, according to a focused and comparative study reported previously [117]; however, it trails behind chitosan, which is also well-known in this regard [118,119]. A pullulan covering mixed with an ethanol extract of bee propolis delayed ripening and weight loss in blueberries, reduced microbiological contamination, and extended shelf life [120]. To date, few studies have focused on the impact of the mixture of pullulan-based edible coatings, anti-browning agents, and antibacterial agents for minimally processed products to increase their shelf lives [121,122].

#### 2.2.5. Xanthan

The industrially important exopolysaccharide xanthan is obtained from phytopathogenic bacteria of the genus Xanthomonas (mostly *X. campestris* pv. campestris), is made from different monomers (glucose, mannose, and glucuronic acid with acetate or pyruvate group), and has gained widespread recognition for its excellent miscibility, rheological properties, and consistency under a wide range of external conditions [123]. A study by Li et al. in 2017 showed that xanthan alone can also be used as an edible coating for fruits such as strawberries and blueberries, hence increasing the shelf life of products coated with substances such as pullulan [117]. A targeted study comparing xanthan with other compounds as edible coatings for blueberries found a significant advantage of this exopolysaccharide [124] over the plant-based guar gum and gum Arabic, which are substantial ingredients in related fruit coatings [119,125,126]. Totad et al., in his studies in 2019, stated that xanthan reduces water loss and retains fruit firmness [124]. It also maintains the level of antioxidants, ascorbic acid, total phenols, and anthocyanin content. Its gelling and film-forming properties can be improved with the addition of lipids [127].

#### 2.2.6. Gellan

Gellan, as with xanthan, is a kind of extracellular heteropolysaccharide generated by Sphingomonas bacteria. Gellan has a good film-forming capacity since it is soluble in water, insoluble in ethanol, and has excellent colloidal and gelling characteristics. However, it has been reported that an edible strawberry coating made of gellan with glycerol as a plasticizer, enriched with natural antimicrobials (particularly geraniol) significantly reduced microbial counts (mesophilic and psychrophilic bacteria, yeasts, and molds), improving the microbiological stability of the berries when compared to untreated samples [128].

#### 2.2.7. Milk Protein

Milk proteins are of two types; caseins and whey proteins. Films made from milk proteins are flexible and transparent in nature. These films also carry active antimicrobial and antioxidant agents to enhance the quality of food. Films from casein are stable at different pH, temperature, and salt levels. These films can act as carriers for antioxidants, food coloring, or antibacterial compounds (food additives). To prepare these films, an aqueous caseinate solution is made followed by drying [129]. Whey protein is obtained by the precipitation of casein protein from milk. Compared to films formed by casein protein, whey protein films show more barrier properties as suggested by Mohamed et al. in his studies [129]. An antifungal whey-based film, which was resistant against *Aspergillus niger* (103 spores/mL), was prepared by Braber [130] and his colleagues. Chitosan was incorporated in low quantities and, as a plasticizer, glycerol was utilized. In order to neutralize the chitosan charges, sodium tripolyphosphate was added. Interestingly, the results showed that whey-based films can be formed with excellent water permeability and good flexibility if almond oil is incorporated into the film formation matrix [131]. Due to its barrier properties, a whey protein coating with oregano essential oil is applied to chicken breast meat to control the growth of spoilage-causing bacteria [132].

### 2.3. Other Materials

#### Lipids

Lipids are not exactly considered to be biopolymers and hence are not suitable for the formation of cohesive films and coatings for food. However, lipids serve as an outstanding barrier against moisture and they can therefore be used to make films and coatings in combination with polysaccharides and proteins, as emulsified particles or multilayer coatings with enhanced characteristics [15]. Lipids are able to block moisture due to their low polarity, and their ability to form films and coatings with other materials depends on the chemical arrangement, chemical structure, chain length, physical state, degree of saturation and hydrophobicity of molecules [133]. The incorporation of lipids in edible films and coatings not only enhance their barrier properties, but also enhance their flexibility, cohesiveness, and hydrophobicity, which thereby improves the sensory properties of foods such as aroma and freshness, appearance, and tenderness. The moisture-blocking property of lipids in edible films can improve the microbiological stability of foods which helps to increase the shelf life of foods [134].

Lipid-based compounds mainly consist of herbal waxes, surfactants, and acetylated monoglycerides, which act as protective coverings on food surfaces [24]. Paraffin wax, a lipid-based compound containing strong hydrocarbon mixtures, is obtained by fraction distillation from crude petroleum. It is a result of the catalytic polymerization of ethylene. Films and coatings made from paraffin wax have found potential applications for covering highly perishable foods such as fruits and vegetables, cheese, etc. Candelilla, carnauba, and beeswax are also used to form coatings on different food products as they can effectively block moisture and vapor and can also improve the appearance of food products [24].

The reaction of acetylated glycerol with acetic anhydride produces 1-stearodiacetin. This acetylated monoglyceride can be easily solidified into a bendy, wax-like solid from its molten state. Acetylated glycerol monostearate has ultimate flexibility as it can extend up to 800% of its length. These films and coatings are usually applied on chicken and meat cuts to inhibit moisture loss during storage [24].

## 3. Functional Material for Edible Films and Coatings

The development of packaging in the form of coatings and films made from food-grade biopolymers has progressed dramatically in the previous decade, resulting in a succession of discoveries that have led to the invention of a variety of edible packaging. When used on food goods, this form of coating, in addition to maintaining the stability of the products, also results in an improved product appearance by minimizing physical damage and scars and by improving the shine of the surface [135]. This coating has the ability to act as a barrier at the product surface, preventing moisture loss, gas smells, and solute movement, as well as offering some functional qualities to the finished product [136]. During film preparation, the following ingredients are incorporated, which are gradually released into the food items, thereby improving their physical and chemical qualities. Antimicrobials and antioxidants, when integrated into films and coatings, enable for the extension of food shelf life and protection against unfavorable phenomena such as oxidation, rancidity, degradation, and discoloration. Furthermore, nutrients, flavors, and colorants can be utilized as active agents to improve the nutritional content of food, by adding vitamins and minerals, as well as the appearance and taste of the food [137]. Some examples of functional materials which are incorporated into biofilms and coatings as reinforcing agents/additives are discussed below.

### 3.1. Nanomaterials

In food packaging, the term “nanoparticle” refers to a wide variety of materials including (but not limited to) carbon materials [138,139], metals [140,141], metal oxides [142,143], mixed metal oxides [144], and nanolayers [145,146,147]. These materials are used as nanocomposites with characteristic physical, chemical, and biological properties [89,148,149].

The purpose of nanoparticles is to improve the sturdiness, abrasion resistance, and efficiency as a moisture, water, light, and environmental gas barrier in standard polymers [150]. Several nanocomposites with improved functions, such as gas, temperature, and humidity resistance, mechanical strength, and flexibility, have been created using various combinations of polymers and nanofillers [151]. Inorganic nanomaterials such as clays, layered double hydroxides (LDH), layered silicates, salts, metal oxides and metallic NPs, cylindrical metal oxide nanotubes (NTs), or gold nanorods (AuNRs) are commonly used as nano reinforcements. Natural NPs such as cellulose nanocrystals (CNCs), cellulose nanoparticles (CNPs), zein NPs, and others have also been used in a variety of polymers. Organic nanoparticles are used for the targeted drug delivery of nutraceuticals in food. Three types of organic NPs are identified: lipid-based, polysaccharide-based, and protein-based [152,153,154]. These nanoparticles aid in the storage of food and the preservation of its freshness and quality [150].

Though nanotechnology in food science has been extensively developed it has yet to be adopted at a large scale, owing to concerns about health and the environment, particularly during the storage stages. Furthermore, due to the multifaceted interaction profiles, detecting nanomaterials in food products (whether released or reacting with food content) is still a difficult task [155,156]. Researchers are still working to incorporate nanoparticles along with other materials to enhance the properties of films. In 2019, Amjadi et al. formulated an active packaging material using chitosan nanofibers with zinc oxide (ZnO) nanoparticles [157]. Cheese was used in their work as the food model to test the efficiency of this bio-nanocomposite film. According to the data, active packaging drastically reduced the growth of inoculation bacteria. Jafarzadeh et al. used ZnO-NPs in semolina films in another study. Antimicrobial activity was found against *E. coli*, *S. aureus*, *Candida albicans*, and *Aspergillus niger* [158]. The antibacterial activity increased when the number of ZnO-NPs was increased. Antibacterial activity was also higher over Gram-positive bacteria than against Gram-negative bacteria. They also showed that these films may be used to package mozzarella cheese. According to Alizadeh-Sani et al., combining TiO_2_ with rosemary essential oil (REO) to make cellulose nanofiber (CNF) films increased antibacterial activity against Gram-positive bacteria such as *S. aureus.* L. monocytogenes against Gram-negative bacteria such as *E. coli O157:H7*, *E. coli O157:H7*, *P. fluorescens* and *P. enteritidis* [159].

### 3.2. Natural Bioactive Material

Bioactive materials are biomaterials that include active chemicals with antibacterial or antioxidant capabilities. Natural bioactive elements are added into films to strengthen their characteristics, hence enhancing methods for food preservation [160,161]. Animal and plant materials from various food industries, such as the fruit and vegetable, grain-processing, brewery and winery, dairy, marine, and meat industries, can be classified into bioactive components from food by-products based on their origin. Each sector generates a considerable amount of residue, including seeds, pulps, peels, leaves, and stems from plant by-products, and skin, bones, and shells from animal by-products, all of which contain valuable compounds such as phenolics, flavonoids, phytosterols, peptides, and other antimicrobial and antioxidant compounds. The fruit industry generates a variety of by-products that are high in phenolic acids and other phenolic compounds, carotenoids, or vitamins, all of which have antioxidant properties. The hydroxyl groups and conjugated double bonds in phenolic substances serve as powerful antioxidants, scavenging free radicals. In addition to scavenging free radicals, phenolic compounds chelate transition metal ions, recombine radicals, and act as electron transfers, resulting in stable products. As a result, plant extracts containing phenolic compounds with significant antioxidant activity are ideal candidates for the development of active food packaging. Many extracts from food by-products demonstrate antibacterial activity against food-related microbes in addition to their antioxidant activity. For example, pomegranate peel extracts (20 mg/mL) inhibited the growth of a variety of food-related bacteria, including *Staphylococcus aureus* and *Bacillus cereus* [162].

### 3.3. Essential Oils (EOs)

Plant-based essential oils have become a popular research topic in recent years because they include functional and active chemicals. These are extracted from different plant parts such as the stems, leaves, flowers, and roots, and they consist of aldehydes, monoterpenes, flavonoids, isoflavones, carotenoids, alkaloids, phenolic acids, terpenes, and aromatic and aliphatic compounds. They are hydrophobic in nature, with a distinct odor [163,164,165]. EOs are extracted from plants as a product of secondary metabolism. They are highly lipophilic, volatile, and prone to oxidation and thermal degradation. Due to their lipophilic nature EOs are immiscible in water.

Because essential oils are derived from natural sources, they are typically considered safe for human consumption and are regarded as GRAS (generally recognized as safe) by the Food and Drug Administration of the United States (US-FDA). Despite the fact that most essential oils are regarded as safe, their usage as food additives is restricted due to their strong aroma. EOs are generally added into films by entrapping or encapsulating them within the polymeric matrix, resulting in decreased volatility and thereby increased efficiency [166]. Recently, numerous studies have showed that the use of EOs in edible films and coatings can enhance their barrier and mechanical properties [167]. Cai et al. added thyme essential oil to starch film to improve its tensile strength and water resistance [168]. Martins et al. found that the incorporation of oregano essential oil increased the tensile strength and elasticity of rice starch films but reduced the solubility and water vapor permeability of the film in water [169]. Nisar et al. added clove essential oil to the citrus pectin film, which improved the water resistance of the composite films [170].

## 4. Fabrication of Edible Packaging Materials

In recent years, the majority of edible component research has been concentrated on composite or multicomponent films in order to better understand the essential benefits of each component while minimizing their drawbacks [171,172]. Composite packaging can be defined as the combination of more than one kind of packaging biomaterial to improve the characteristics of overall packaging. The primary goal of composite (heterogeneous) films and coatings is to improve permeability or mechanical qualities [15]. Polysaccharides and protein-based films have high gas barrier capabilities, but they demonstrate an insufficient water vapor barrier. Lipids, on the other hand, have a good water barrier but a bad gas barrier. That is why composite films are made by combining lipid-based materials with polysaccharides or a protein-based polymer matrix in order to enhance gas and water vapor barrier in the resulting films. Lipids in composite films may also help to increase water vapor and oxygen barrier characteristics [15]. The composite coatings can be formed as either bilayer or stable emulsions [24]. The lipid forms a second layer above the polysaccharide layer in bilayer coatings. The lipid is disseminated and entrapped in the supporting matrix of protein or polysaccharide in emulsion coatings [173].

The efficacy of lipid material in this type of coating is determined by the lipid structure, chemical arrangement, hydrophobicity, physical state, and interaction with the other components of the film. Four processes are involved in the preparation of bilayer films and coatings: two casting stages and two drying stages. Several studies have documented the advancement of composite edibles and coatings. These studies include the use of lipid and hydroxypropyl methyl cellulose composite coatings, methyl cellulose (MC) and lipid composite coatings, corn zein, methylcellulose and fatty acid composite coatings, gelatin and fatty acid composite coatings, gelatin and soluble starch composite coatings, and corn zein and corn starch composite coatings [24]. Table 2 summarizes the brief details regarding the biopolymer-based films and coatings for food packaging applications.

**Table 2:** Brief summary for biomaterials used in edible packaging.

Biomaterial	Source/Derivative	Properties	Applications	References
Flour of grains and legumes	Corn starch, chickpeas, lentils, etc.	High content of starch and protein results in good film-forming properties.	Used as an edible film for wrapping foods.	[13]
Fruit and vegetable residues	Peel, pomace, seed fraction, etc.	Films are malleable, water soluble, improved mechanical and barrier properties, enhanced mechanical resistance.	Can be used as edible films and coatings for perishable food items.	[13]
Plant gums	Gum arabic, gum karaya, gum ghatti, mesquite, etc.	Potential material for edible film formation, exhibits good physical, chemical, biological properties.	Edible films and coatings on fruits such as strawberries, tomatoes, and pecan nuts.	[13]
Pectin	Cell wall of plants	Acts as a natural plasticizer, good mechanical properties, poor barrier properties.	Edible coating for fruits, vegetables, cheese, and meat products.	[24]
Starch	Corn, potato, wheat, rice, etc.	Tasteless, clear, O_2_ and CO_2_ barrier, not soluble in water, poor mechanical properties.	Wrapper and coatings after some modifications.	[174]
Cellulose	Carboxymethyl cellulose (CMC), cellulose acetate (CA), methyl cellulose, ethyl cellulose, hydroxypropyl, hydroxyethyl cellulose, cellophane, etc.	Not moisture resistant, good mechanical properties.	Used for packaging of meat products, confectionary, and cheese.	[77]
Protein	Wheat gluten and corn zein, soy protein	Water solubility, opacity, and mechanical and barrier properties, Not soluble in water, but absorb water when they are submerged.	Can be used after modifications, used in glues, dyes, and paper coatings.	[24,76]
Marine algae	Alginate, agar, carrageenan	Natural binder in coating. Poor water resistance, good barrier for oxygen, lipid, and oxidation of fat.	Edible coatings for fruits, vegetables, cheese, and meat products.	[77]
Chitosan	Exoskeleton of crustaceans, fungal cell walls, and other animal sources	Good mechanical properties, good barriers for oxygen transition, antimicrobial properties.	Used in fruit coatings, cellophane packaging.	[77]
Collagen and gelatin	Hair, skin, nails, bones, and ligaments of beef or fish	Translucent films, permeable to moisture.	Used in the coating of sausage and other meat products.	[29]
Pullulan	Microbiological source	Thermal and oil resistance, low gas barrier.	Used for food coatings.	[175]
Xanthan	Microbiological source	Excellent solubility, good rheological properties and stability.	Used in coatings for fruits such as strawberries, garden berries, and grapes.	[176]
Gellan	Microbiological source	Exhibits good gelling, colloidal, and antimicrobial properties, exhibits good water solubility.	Used in edible films and coatings for fruits and vegetables.	[176]
Milk proteins	Caseinate and whey protein	Casein-based films are opaque and water insoluble, but they absorb water, and have good mechanical properties; expensive.	Can be used to coat highly perishable foods such as meat products.	[177,178,179,180]
Lipid	Acetoglycerides, beeswax, surfactants, triglycerides, fatty acids	Weak mechanical properties, good barrier against moisture migration.	Edible coatings for fruit and meat products.	[77,181,182]
Natural bioactive material	Fruit waste, agricultural waste (antioxidant and antimicrobial compound)	Exhibits excellent antioxidant and antimicrobial properties.	Can be used as an active agent in edible films such as agar-based films.	[29]

## 5. Fabrication of Composite Films and Coatings

While creating edible films and coatings from different biomaterials, it is important to keep in mind the ability of films to adhere to food surfaces and the methods by which edible films and coatings should be applied to food products so that they do not deteriorate the quality of the food [183]. The difference between the application of edible films and coatings on food is that films are formed separately by different methods and then applied, whereas coatings are directly applied to the food materials. The simple or mixed usage of diverse carbohydrate, protein, or lipid components in various forms (coatings, single-layer, bilayer, or multilayer films) has been proposed for the manufacture of edible films and coatings with regulated barrier properties and is acceptable for high-moisture foods [184].

### 5.1. Film Fabrication Methods

Gontard et al. (1996) proposed the following mechanisms of edible films formation after his studies on wheat gluten films [185].

1. Simple coacervation: This occurs when a hydrocolloid dispersed in water precipitates or changes phase after solvent evaporation (drying), after the addition of a hydro-soluble non-electrolyte in which the hydrocolloid is insoluble (e.g., ethanol), or after pH adjustment and the addition of an electrolyte that causes salting out or cross-linking.
2. Complex coacervation: When two hydrocolloid solutions with opposite electron charges are combined, the polymer complex interacts and precipitates.
3. Gelation or thermal coagulation: This is a process in which a macromolecule is heated, creating denaturation, and then gelatin (e.g., proteins such as ovalbumin) or precipitation, or even the cooling of a hydrocolloid dispersion, causing gelation (e.g., gelatin or agar).

Edible films can be used as primary edible packaging materials by wrapping them around the food surface. The solubility of additives in biopolymers is a critical aspect in their effectiveness. The overall mechanical properties of the film are influenced by the cohesive forces between biopolymers. Different methods for film deposition on food products have been investigated by scientists. Film-forming biopolymers and additives should have their chemical and structural properties well understood and modified for the generation of films for specific applications [186]. Orally soluble films containing active ingredients have been generated as a result of recent technological advancements in edible film fabrication. Ingredients for oral hygiene, caffeine for alertness, minerals, and botanicals are examples of actives included in film strips. (Bilal et al., 2020). Suhag et al. (2020) discussed two methods for obtaining edible films from bio-based materials [187].

#### 5.1.1. Solvent Casting Method

**Figure 3:**
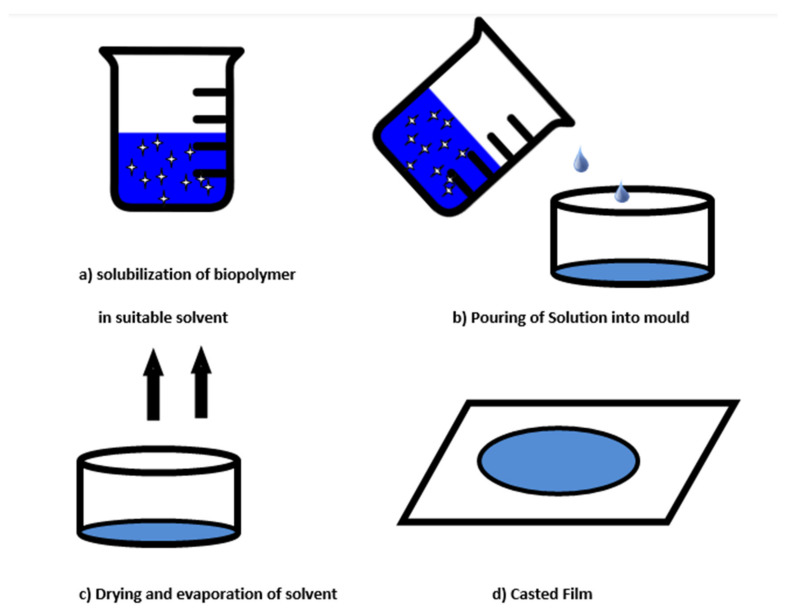
Step-by-step showing fabrication of edible films by solvent casting method.

The casting process is a popular and inexpensive method of film preparation. To make a biopolymer film, this process entails three phases as presented in Figure 3 [188].

(i) Solubilization of biopolymer in a suitable solvent: Selection of suitable biopolymer for film formation is the first basic step in edible film formation. The selected polymer or mixture of polymers is now dissolved in suitable solvent and this step is known as solubilization. For example, ethanol is used to dissolve soy protein isolate polymer. The casting of the film is dependent on the solubility of polymer rather than a melting process [189,190].

(ii) Casting of the solution in the mold: In this step, the resulting solution is poured into a predetermined mold or Teflon-coated glass plate. The drying process allows the solvent to evaporate, resulting in a polymer layer that adheres to the mold.

(iii) Drying of casted solution: To enhance the intramolecular interaction between the polymer chains and obtain a proper microstructure of the film, the air-drying technique for casting edible film is critical [191]. Rapid drying of the casting solution should be avoided because it reduces the solvent concentration too quickly, restricting polymer chain mobility and the formation of intermolecular interactions in the film [181]. For the casting of films, air driers such as hot air ovens, tray dryers, microwaves, and vacuum driers are used to remove solvents and peel the film [192].

The film’s structure is determined by the composition of the casting solution, the thickness of the wet casting, and the temperature and relative humidity of the drying circumstances [181]. COGIN, Bio Envelop, Chris-Kraft Polymer Inc., GREENSOL, and ENAK are among the companies that use this approach to produce edible films on a commercial scale [187]. Because it is a wet process, the bonding between the molecules of biopolymer is strong, which results in homogeneous coating. Since the casting method is a low-temperature production method, there is no risk of molecular degradation as a result of temperature fluctuations [15].

The main advantage of the casting method of film production is the ease with which it may be manufactured without specialized tools and at a reasonable cost at a lab scale (Chen et al., 2008). Because most food-processing materials cannot be molded at higher temperatures without causing irreversible structural changes, the lower temperature during the processing steps is another advantage [187].

The following are the major limitations of the casting method: (i) limiting the forms (usually, the only forms that can appear are simple sheets and tubes); (ii) casting requires a long drying time, which is not feasible for commercial production [189]; (iii) one of the most difficult challenges is converting film production from laboratory to production scale because many variables, such as heating, and the combination of speed and temperature, can cause quality differences and prevent constant development for commercial scales [193].

#### 5.1.2. Extrusion Method

**Figure 4:**
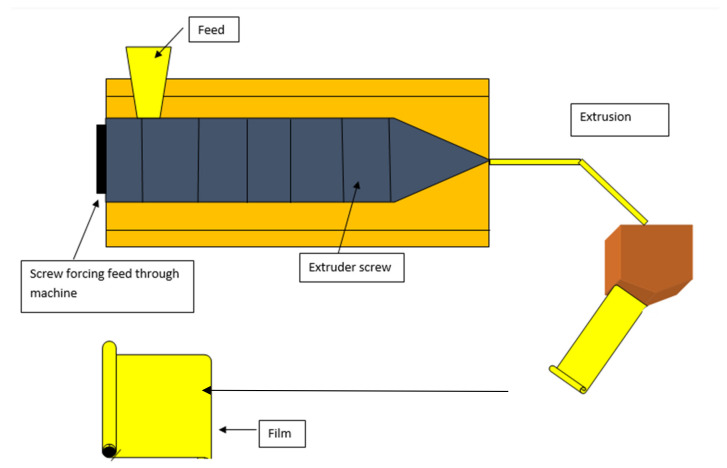
Extrusion technique used for the formation of films.

The extrusion process is based on the polymers’ thermoplastic characteristics [80]. In general, the extrusion process can be separated into three zones as shown in Figure 4 [194].

(i) The feeding zone: The film component mix is first carried into the feeding zone and then compressed with air, then the biopolymer and additives are added to the extruder.

(ii) The kneading zone: Wherein materials are adequately combined with the help of an extruder screw.

(iii) The heating zone at the final part/exit from the machine: The oven is used to supply some heat in the heating zone. Here, biopolymer and additives are melted and mixed. A die is fixed at the extruder’s end, determining the shape and thickness of the extruded film.

Because this approach works best with a small amount of water or solvents, it is also known as a dry procedure. Plasticizers, on the other hand, are required to increase film flexibility [195]. Plasticizers such as polyethylene glycol or sorbitol are frequently extruded in amounts ranging from 10% to 60% by weight [181]. Due to the lack of solvent addition and evaporation phases, extrusion is preferable in commercial applications.

We can make a multilayered film with better overall qualities if we utilize more than one extruder. Co-extrusion is the term for this procedure. In comparison to single-layer extruded film, the finished co-extruded film will have combined and improved qualities. Mechanical, optical, and barrier problems can occur as a result of variances in the chemical–physical characteristics of each film-forming substance [181].

The advantage of the extrusion method is that it is a high-performance, low-cost, and efficient process that is employed in the food industry for commercial manufacturing [25,196]. Other benefits include the absence of solvents, the ease of the handling of high viscosity polymers, a wide range of processing conditions (temperatures of 70–500 °C and pressures of 0–500 bar), and the improved control of feed residence time and mixing degree [197]. The comparison between the two film-making methodologies is presented in Table 3.

## 6. Methods of Coating

The efficiency of a coating depends on the method used to coat fruits, vegetables, and other foods [25,198]. The techniques used for this (dipping, spraying, etc.) are determined by the type of the food to be coated, its surface qualities, and the coating’s objective. The adhesion process, which involves diffusion between the surface area of the food product and the coating solution, follows the coating operations [135,199]. Surface tension, density, shape, size, coating thickness, and other physical factors, as well as food product attributes, influence coating methods [200,201]. Coatings are applied on food in the form of liquid suspensions, emulsions, powders, and other forms. Drying, heating, chilling, and coagulation processes are used to change the covering layers of food ingredients [187].

### 6.1. Dip Coating

Dipping is the most popular way of coating a food product, which involves forming a thin film on the surface that works as a semipermeable membrane to control moisture loss and gas transmission (Lin and Zhao, 2007). Food products are dipped in a prepared film formation solution to cover the surfaces of fruits, vegetables, and meat products in this method. This process is divided into three phases [82,202].

Immersion and dwelling: This involve immersing the substrate at a steady speed in the prepared coating solution to ensure complete interaction between the coating solution and the product’s surface.

Evaporation: The component’s coating composition must be diluted. After dilution, a considerable amount of residual material of the coating is formed. After the solvent has evaporated and the extra liquid has been removed, a thin film is produced [200].

Deposition: In this step, the precursor solution of the thin layer is formed on the surface of food products such as fruits, vegetables, and meat products by deposition.

In a past study, dipping techniques were utilized to improve the shelf life and appearance of fruits and vegetables, as well as other food products [203,204,205]. Because the dipping procedure is usually relatively short, the evaporation of solvents from the coating and crosslinking solutions is overlooked. The dipping and draining times vary by study, although they usually last 30 s to 5 min. The method’s key benefit is that it coats the entire surface, even if it is intricate or rough [135].

The dipping method is extensively used to coat fresh vegetables with edible coatings. In general, fruits and vegetables are submerged for 5–30 s in the edible coating formulation, and most fruits are simple to coat [25]. Dipping freshly cut fruit in an antimicrobial-containing aqueous solution is the most effective technique to increase microbial stability. The most frequent way to prevent fresh fruit from browning is to use antioxidant treatments as dips after peeling and/or cutting [206]. Chitosan coating is carried out on frozen salmon fish using the dipping method. This coating prevents pathogenic bacteria from growing and extends the shelf life of the fish [207].

### 6.2. Spray Coating

The spraying method is commonly utilized in industries on a broad scale due its low cost and high quality of end product [208]. In this method, a liquid solution is applied onto food products by spraying.

**Figure 5:**
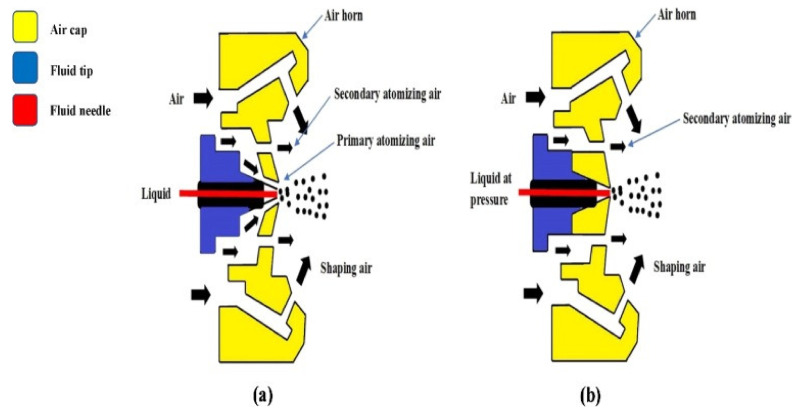
(**a**) Air spray atomization, (**b**) Air-assisted airless atomization [187]. (Reprinted with permission).

The liquid solution is converted into small droplets when it is sprayed. These droplets will have larger surface area for the same amount of liquid solution. As a result, droplets will cover a larger portion of the substance [187]. With the use of nozzles, the spraying system distributes the coating solution by forming droplets over the specified food surface area. Because of the high spraying pressure (approximately 60–80 psi), the spraying technique requires less coating material to obtain effective coverage [135]. Furthermore, a thickness of 30 μm was best for guaranteeing that water vapor and mechanical qualities were maintained, and therefore this parameter must be carefully controlled [209]. This approach can be classified into the following types based on how the droplets form (Figure 5).

Air spray atomization: A high-velocity stream of air surrounds fluid flowing from a low-velocity tube in this spraying method. Fluid-air friction causes atomization by speeding up and disrupting the liquid fluid flow. A spray is created through the nozzle. To break up the cylindrical water jet into fine droplets, a cylindrical water jet (deflector) air jet is utilized. It is mostly utilized for a fine droplet spray on food and food goods. Due to the use of air for spraying and the reduction of water volumes for product coating, it is a cost-effective technology [187,210].

Airless atomization: Because the edible coating is applied to food goods using pressure rather than air spray atomization, it is also known as pressure atomization. High pressure through a small nozzle is used to apply the coating solution to food goods. The pressure is a critical parameter that must be kept below 3.5 bars to avoid the destruction of the film-forming mechanism [200].

Air-assisted airless atomization: This approach combines the benefits of both airless and air spraying. Because of the combination of both, it gives great efficiency and creates droplets. With a unique fluid nozzle tip similar to a regular airless tip, air-assisted airless spray guns partially atomize the fluid. Second, they employ small volumes of compressed air from the face and/or horns of the air nozzle they use to finish the atomization. As a result, the spray pattern is finely atomized and resembles that of a compressed air system. This technique’s gentle spray helps to reduce fog and waste. This method is both novel and effective for edible coatings on food goods, as well as for improving the preservation efficiency of fruits and vegetables [208].

The pressure, viscosity, surface temperature, and tension of the coating solution, as well as the spray nozzle shape and design, all affect spraying efficiency. The nozzle shape affects the flux rate, droplet size, distance, angle, and overlap rate [201]. The spray piston and nozzle, as well as the temperature, air, and fluid flow of the polymer, all have a role in controlling the final drop size and hence the coating quality [211]. The uniform coating, thickness control, multilayer application capability, avoidance of coating solution contamination, solution temperature control, and ability to deal with vast surface areas are all advantages of this technology. The fact that excessively viscous biopolymer cannot be sprayed is a major disadvantage of this approach. The dipping approach is preferred for high-viscous biopolymers [187,200].

### 6.3. Electro Spraying

Traditional coating processes result in high coating-material losses (about 50%) and a heterogeneous coating material distribution on the product surface. That is why a coating process that can efficiently deposit the film uniformly over uneven surfaces is required. Electrostatic coating, which originated from the paint industry, is a promising and effective coating approach for foods. It is a more advanced version of the traditional spraying approach that increases the effectiveness of liquid particulate transfer to food particles. Since it has a higher transfer efficiency and it is able to form reproducible coatings on a target surface, it is applied to food products to reduce the use of solvents and the production of waste. In addition to the paint industry, this technique is often applied in the pharmaceutical and automotive industries. Based on the type of coating material, this technique can be divided into two types: dry (powder) and wet (liquid) coating [212].

#### 6.3.1. Electrostatic Powder Coating

This method was previously utilized in the automotive painting industry in 1960, before it finds its application in the snack food processing industry [213]. When compared to non-electrostatic coating, this process produces a more even, uniform, and reproducible coating with less waste or overuse of expensive food powders because the powder particles are charged and repel one another, resulting in an even dispersion throughout the target surface. During powder coating, wire electrodes in the coating chamber or at the end of a charging gun create an electrostatic field. The charging of powder by an electric field is the initial stage in electrostatic coating. Charged particles resist each other due to their identical charge, generating an even cloud across the target surface [214]. The powder is more broadly dispersed and has a higher transfer efficiency at higher charges [215]. Two mechanisms can be used to charge powder: corona charging and tribo-charging. Powder particles pass through an ion-rich zone and are charged based on their permittivity in corona charging. As a result of the charge difference, they are transported towards the target and deposited. Tribo-charging is another method that uses the frictional charge of powder that is delivered through a pipe made of a certain material, such as PTFE, metal, or other powder particles [213,216,217]. Because tribo-charging leaves less charge on particles than corona charging [218], corona charging is the preferred method for electrostatic powder coating [219]. When a pneumatic system is utilized, the air movement produces aerodynamic force in addition to gravity and Coulombic forces, which transports the charged powder particles from the dispenser to the target surface [213]. The coating performance, including transfer efficiency, dust, adhesion, evenness, and the functionality of the electrostatically coated product, is influenced by the physical properties of the powders, including particle size, shape, charge, flow characteristics, resistivity, and density, as well as the target properties. Food powder coating is used in several food processes, particularly in the snack food industry, to improve the appearance and flavor of products such as potato chips, cakes, doughnuts, pretzels, crackers, and shredded cheese, resulting in increased market acceptability [220].

#### 6.3.2. Electrostatic Liquid Coating

Electrostatic coating, also known as electrohydrodynamic coating, can be used to apply liquids. This is one of the most important developing technologies for applying oils, flavors, and emulsions to foods [221]. A liquid is dispersed into fine droplets, ranging in size from 0.1 to 1000 mm, in this coating technique, where the electrostatic force charging the liquid’s surface causes the stream of liquid to split into droplets [222]. In electro spraying, the coating substance is charged while travelling through a nozzle attached to a high-voltage source. These charges (electrostatic forces) break up the liquid jet into micro-droplets by overcoming the liquid’s surface tension. These forces also cause the liquid to shear, resulting in a fine cone-jet [223]. Charged micro-droplets resist each other in the air, creating a liquid cloud. With the charging of droplets, the coalescence of droplets is eliminated, and uniform-sized droplets are created. These charged droplets are attracted to the grounded surface with a high transfer efficiency (about 80%) and release their charge after deposition [224,225].

Electro spraying has also been used to produce nanoparticles of various proteins (such as WPI, WPC) and folic acid in starch, which improves the characteristics of these materials [226,227,228]. Similarly, probiotics with homogeneous particle size and a high number of viable counts have been encapsulated [229,230]. Proteins, carbohydrates, and lipids, either alone or in combination, serve as encapsulating materials (in the form of gel or solutions).

### 6.4. Panning Method

**Figure 6:**
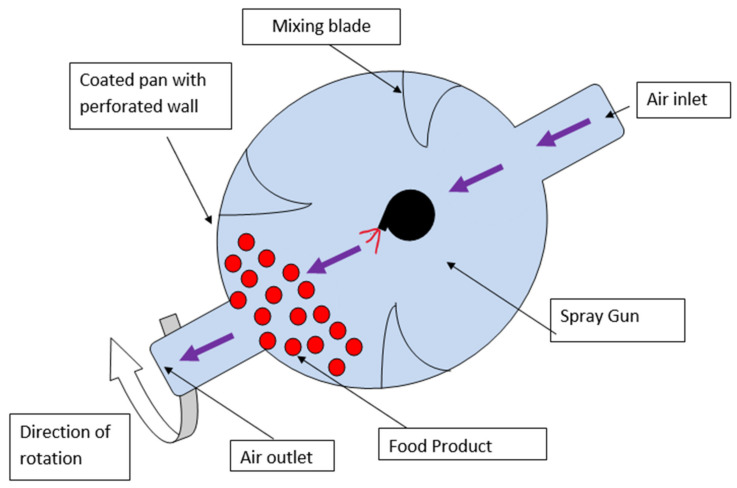
Panning method of edible coating.

The panning coating process was developed within Greek Arabian society and was first utilized for drug applications. The panning method consists of placing the food and other items to be coated in a large rotating bowl known as a pan (Figure 6). The coating solution is then drizzled or dusted on the surface of the food product in a spinning bowl, and the product is tumbled to evenly distribute the coating solution. Coating layers are dried using forced air at room temperature or higher [231,232]. Heat is generated by friction with cold air during the panning process of applying coating to food products. This process is ideal for coating foods and confectionary. This approach is able to coat a large number of round or oval-shaped food items in a single batch.

The pharmaceutical industry has recently used a revolutionary electrostatic dry powder pan coating procedure to coat the surface of capsules and tablets. It is a well-established and widely used technology for improving powder deposition quality and coating uniformity. It is a good approach for coating conductive materials with the coating material. A powerful electrostatic charge is applied to the substratum. The substance composed of the leading ion species of the opposite charge is sprayed on the charged substratum. Fully and uniformly covered corners are obtained on the substratum [187].

### 6.5. Fluidized Bed Processing Method

The food sector initially rejected this technology due to the high costs involved. When compared to other coating technologies, the fluidized bed requires a greater amount of coating solution due to the loss on the column wall during spraying. In food processing and research, the fluidized bed method of edible coating is frequently utilized. It is used to coat dry particles with thin layers of coating material that are extremely low density and/or small in size. In a fluidized coating process, the coating solution and suspension are sprayed onto the fluidized powder surface via a number of nozzles to form a shell-like structure. When a flow of liquid travels upward through a bed of particles at a sufficient speed to assist the particles without diverting them into the liquid stream, the process is known as fluidization. At this moment, the particle bed accepts the bubbling fluid’s characteristics, i.e., fluidization [200]. There are three types of fluidized bed processes: top spray, bottom spray, and rotating fluidized bed. However, in the food industry, traditional top spraying is more effective than other methods [233]. Because the powders in the conventional bed do not fluidize stably or form excessive agglomerations in smaller sizes, the particle matter in the fluidized bed should be larger than 100 μm [234,235].

Fluidized bed coating prevents the creation of coated product clusters, which is a typical problem in pan coating. Fluidized bed coating also displays good drying effectiveness and allows for the use of a smaller surfactant choice in comparison to dipping and panning forms [236]. It does, however, take less time to process, gives complete coverage, and reduces cluster formation [237].

## 7. Application of Bio-Based Films and Coatings in Food

The above-mentioned film forming materials and methods for the creation of films and coatings have important applications in the food sector. When compared to conventional plastic packaging, edible packaging provides a selective barrier for water and oxygen, extending the shelf life of food goods [238]. Films and coatings have different applications, which are briefly covered in the sections below.

### 7.1. Application of Films

Edible films are wrapped around the food surface and used as the principal edible packaging for perishable foods such as meat, poultry, and dairy. The application of bio-based film in food packaging is briefly presented in Table 4.

**Table 4:** Application of edible films included with active ingredients and their effects on food products.

Biomaterial	Food Product	Method	Key Results	References
Starch–alginate films with stearic acid	Ground beef patty	Casting	Improved barrier properties against moisture and helpful in preventing oxidation of lipids.	[135]
Chitosan with essential oil	Chicken fillet	Casting	Exhibits antimicrobial and inhibitory activities against spoilage-causing microorganism.	[239]
Carrageenan with olive leaf extracts	Lamb meat	Casting	Good antioxidant activity due to presence of phenol, exhibits antimicrobial activity against Escherichia coli, Coliform. Demonstrates lower water vapor permeability which enhances its shelf life.	[240]
Chickpea with gallic acid	Highly oxidative food	Casting	Antioxidant activity, phenolic content, demonstrates low water vapor permeability and good mechanical properties.	[36]
Gelatin/cellulose nanofibril with Ag nanoparticle and glycerol	Fruits and vegetables	Casting	Good antimicrobial activity against various microorganisms such as *E. coli* and *S. aureus*.	[241]
Corn starch with glycerol	Mango	Extrusion	Able to maintain physical and chemical qualities of mango for up to 16 days at 12 °C.	[242]
Whey protein isolate with oregano essential oil, garlic oil, nisin, natamycin	Kasar cheese	Casting	Effective against Listeria monocytogenes, staphylococcus aureus and Escherichia coli.	[243]
Zein/gelatin with tea polyphenol	Fruits and vegetables	Casting	Antimicrobial properties, prevents browning, and controls weight loss in fruits and vegetables.	[244]
Tapioca Starch/Chitosan Nanoparticles	Cherry tomatoes	Casting	Antimicrobial property against gram positive bacteria, improves shelf life.	[245]
Sodium-caseinate		Extrusion	Good mechanical properties, water vapor permeability, water soluble.	[246]
Sweet potato starch	Baby spinach leaf	Casting	Antimicrobial against *E. coli* and *S. typhimurium*	[247]

Sodium alginate is a brown algae-derived biopolymer based on natural polysaccharides. It is a hydrophilic biopolymer with excellent film-forming properties. It has an excellent oxygen barrier. These alginate films were originally employed to regulate the rate of respiration of fruits and vegetables, but they are now used to wrap meat products. These edible films, when combined with active and antimicrobial ingredients, help to extend the shelf life of meat products [248]. Chicken breast meat is packaged using an alginate-based edible film with black cumin oil added as an antimicrobial agent. When stored at 40 °C, this film inhibits the growth of *Escherichia coli* and the change in meat color for about 5 days.

When compared to polysaccharides and other protein-based films, edible films created from whey protein have greater gas barrier qualities. Whey protein-based films have a higher film-forming tendency. Whey protein is combined with additives such as plasticizer, glycerol, and pH adjustment agents. Whey-protein-based films are similarly transparent, allowing the buyer to see the cheese’s quality [130]. The whey protein matrix contains antimicrobial compounds. Bacteria and yeast will be inhibited by these substances. Antimicrobial agents applied directly to the cheese have a lower likelihood of activating than agents introduced to the whey protein matrix. *Staphylococcus* spp., *Pseudomonas* spp., Enterobacteriaceae, yeast, and mold did not grow appreciably after 60 days of storage at 4 °C when whey protein isolates containing antimicrobials such as natamycin and lactic acid were utilized for the packing of semi-hard bovine cheese [249].

To improve the storage life of strawberries, Mali and Grossmann [250] used yam starch films as packaging. The researchers found that starch films slowed the degradation of stored samples and minimized microbial contamination. In addition, coated strawberries had a 21 day shelf life compared to 14 days for untreated strawberries. The polyphenol and tannin content in pomegranate peel extracts incorporated into zein films (2.5–7.5 g/100 g zein) has been demonstrated to have dose-dependent antimicrobial activity against *E. coli*, *Clostridium perfringens*, *Micrococcus luteus*, *Enterococcus faecalis*, *S. aureus*, *Proteus vulgaris*, and *Salmonella typhii microbial* [251]. Up to 15 days of storage, these antimicrobial zein coatings protected Himalayan cheese from deterioration, as evidenced by a decrease in total bacterial, mold, and yeast counts. In vitro experiments showed antimicrobial activity against *S. aureus*, *B. cereus*, *B. subtilis*, and *E. coli* using aqueous extracts of grape seeds from winery production mixed into chitosan films [252]. These antimicrobial films were applied to chicken breast fillets, resulting in a considerable reduction in the total mesophilic aerobic and coliform bacteria in the product after 15 days of storage and boosting lipid oxidation inhibition.

### 7.2. Application of Coatings

Coatings, which are applied directly to food, act as a barrier against contamination, air, moisture, and other contaminants while also extending shelf life by improving barrier qualities. The application of bio-based polymer coating in food packaging is discussed in Table 5.

**Table 5:** Application of edible coatings incorporated with active ingredients and their effects on food products.

Biopolymer	Active Ingredient	Results	References
Coconut protein and guar gum	Transglutaminase enzyme (oxygen scavenger)	Better barrier properties, improved mechanical properties, water vapor permeability and oxygen transfer rates.	[253]
Chitosan	Ascorbic acid	Suppresses browning activity and prolongs microbial and chemical shelf life of freshly cut apples.	[254]
Starch	*Adiantum capillus-veneris* extract	Helps in preservation and microbial treatment of freshly cut apples.	[255]
Gelatin, chitosan, and cassava starch	*Hibiscus rosa-sinensis* (plant mucilage)	Antimicrobial properties.	[256]
Carboxymethyl cellulose	*Impatiens balsamina* L. extract	Coating on “Xinyu” tangerines shows a delay in ripening, decreased decay rate, increased antioxidant activity.	[257]
Alginate	Green tea extract	Improves safety in strawberries and raspberries against food-borne pathogens.	[258]
	Aloe vera extract (pure and diluted)	Applied on tomatoes; pure extract inhibits the growth of bacteria while diluted extract maintains the quality of tomatoes during storage. It also helps to delay ripening.	[259]
Chitosan	Nano emulsion with lemon oil extract	Increased shelf life of food product up to 7 days.	[260]
Carrageenan	Ascorbic acid/citric acid/ oxalic acid	Coating applied to apples; possess antifungal and antioxidant activities. Improves color and firmness of product.	[121]
Gum Arabic	Lemongrass oil and cinnamon oil	Shows antimicrobial activity on coated fruits such as banana and papaya.	[71]
Candelilla wax	Mineral oil	Prevents weight loss and helps in the prevention of loss of color; retain firmness of fruits such as guava and persian lime.	[24]
Lipid	Stearic acid, polyglycerol polyricinoleate and butter	Provides excellent barrier against moisture in hygroscopic candy tablets.	[24]
Chitosan and pectin	Trans-cinnamaldehyde, beta-cyclodextrin hydrate	Helps in extending shelf life of freshly cut cantaloupe at 4 °C.	[261]
Pectin and sodium alginate	Essential oil	Prevents weight loss, demonstrates antioxidant and antimicrobial activity, improves sensory characteristics in fruits such as raspberries and prevents discoloration.	[57]
Chitosan and alginate	Resveratrol	Antioxidant and antimicrobial activity, prevents oxidation in smoked sea bass fillets.	[262]
Chitosan blended with gelatin	-	Effective antimicrobial activity against *S. aureus* and demonstrated a considerable decrease in count of aerobic and total coliform in perishable foods such as beef.	[263]
Carboxy methyl cellulose	Apple peel (1%) and tartaric acid (0.75%)	Prevents lipid oxidation and shows significant decrease in aerobic plate, yeast, and mold in fresh beef patties. Additionally, no negative impact on sensory characteristics is observed.	[264]
Carboxy methyl cellulose	Ascorbic acid	Improved water vapor resistance, significant reduction in polyphenol oxidase activity, able to retain freshness of carrots during storage period.	[265]
Gelatin and Glucose	Sorbitol and cysteine	Acts as probiotic coating on hake fish due to presence of *Lactobacillus acidophilus* and *Bifidobacterium bifidum*, resulting in enhancing shelf life up to 15 days at refrigerated conditions.	[266]
Cassava starch, chitosan and gallic acid	Glycerol	Increased shelf life of ham slices.	[267]
K-Carrageenan and chitosan	Glycerol and oriental mustard extract	Reduced contamination of *Campylobacter jejuni* in fresh chicken breast along with enhancement of shelf life.	[268]
Hydroxypropyl methyl cellulose and chitosan	Bergamot essential oil	Observes inhibitory action and control over respiration rate and weight loss on cold-stored grapes.	[269]

Research is ongoing in food packaging to incorporate different ingredients into the polymer matrixes, which can add some value to the food product [137]. These ingredients could be any colorant, flavors, nutrients, antioxidants, or antimicrobial compounds [24]. These bioactive compounds act as carriers for active ingredients which allow for the controlled release of functional materials while restricting the undesirable reaction between food and that compound. This kind of packaging is known as active packaging, which enhances the organoleptic characteristics of food while also improving its shelf life and nutritional value [270]. These functional compounds improve the properties of food by interacting with other ingredients by migrating to the middle or onto the surface of food. Their role in coating depends on the morphology, the microstructure and the material of the film, the chemical composition and polarity of the active ingredients incorporated and on the nature of the food product. The safety prospects of this technology are under research since films and coatings may also have a negative impact on food products [271].

The mode of action of these edible coatings involves: (a) the controlled release of active ingredients into food and, (b) the scavenging of undesirable compounds (browning and ethylene) and gases (oxygen and carbon dioxide) from the packaging surface [137].

**Figure 7:**
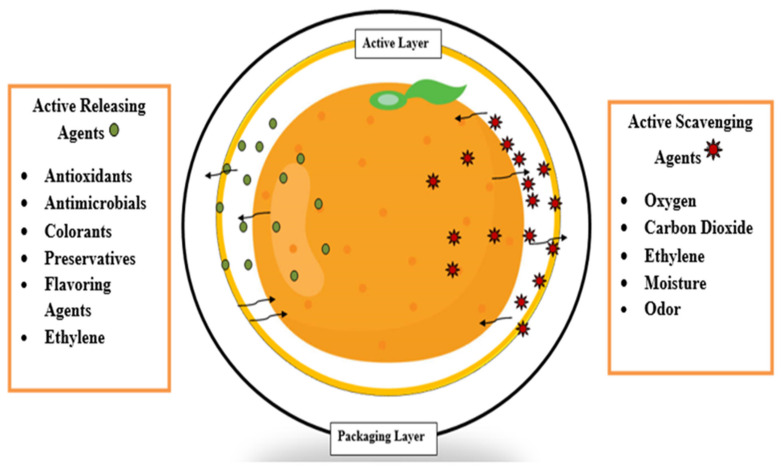
Mechanism of active packaging on a tangerine.

The mechanism of active packaging is shown in Figure 7. When these coatings are formulated, scavenging compounds are incorporated into the coatings. Oxygen scavengers are also used to prevent the browning reaction of fruits and vegetables. The most commonly used synthetic oxygen scavengers are ferrous oxide, catechol, nylon, ascorbic acid, sulphites, and certain other enzymes [137]. These compounds react with the oxygen present in the packaging and decrease its concentration inside the food [272]. Carbon dioxide can also be used as an oxygen scavenging material since it has antimicrobial effects and can also help to overcome the partial vacuum created by oxygen scavengers. Some of the carbon dioxide emitters/scavengers commonly used are calcium hydroxide, potassium hydroxide, silica gel, etc. [272].

Similarly, browning effects can affect the organoleptic properties of food and hence anti browning compounds are cross-linked within a biopolymer matrix which prolongs the shelf life of food products when the active coating is applied to the food surface [273]. Ethylene, produced during the ripening of fruits and vegetables, results in the accelerated ageing of products, leading to deteriorated quality. Potassium permanganate, which acts as an ethylene absorber, can be incorporated into polymer in small amounts since high amounts could be toxic and harmful for health if they come into direct contact with food [137].

In order to increase food safety and prevent contamination, antimicrobial agents are added to films. These ingredients are released by evaporation/diffusion based on their volatility after remaining on food surfaces at high concentrations during storage [270]. The antimicrobial agent selected for this purpose should be compatible with the food product. Chemical agents, including organic acids, ethanol, and metals, and natural agents, including bacteriocins, enzymes, essential oils, and plants extracts, are used as antimicrobial agents in films and coatings. These agents are available in the form of extracts, concentrates, and integrated ingredients [270,272]. Practically, the natural functional agents or bioactive natural compounds are a much safer option over the chemical agents due to their nontoxicity.

In food products, unpleasant flavors and odors occur due to fat and lipid oxidation, which makes food products unacceptable for consumers. It is also responsible for the degradation of polyunsaturated fatty acids and the formation of toxic aldehyde which causes nutritional loss and the spoilage of food product [271]. This oxidation can be reduced by the use of antioxidants, which prevent the formation of free radicals by intruding within the free radical chain mechanism which is responsible for the oxidation process. These antioxidants are incorporated into coatings materials and then applied by coating onto oxidation-sensitive perishable food items such as meat and poultry products [270].

**Figure 8:**
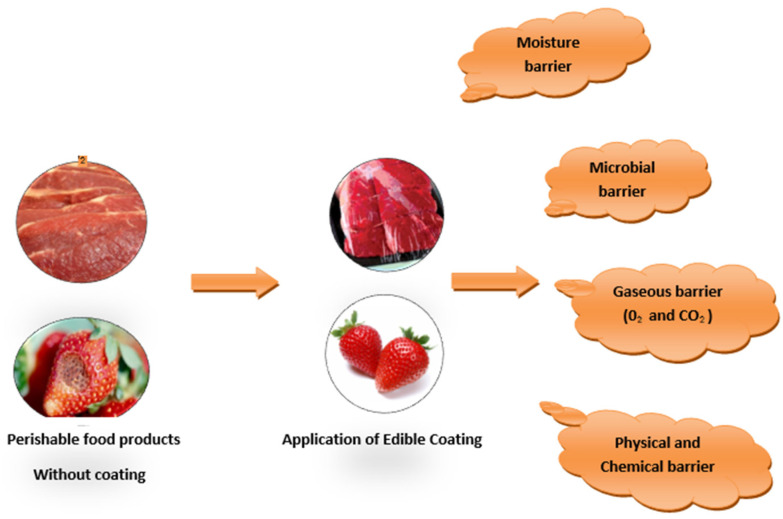
Application of edible coatings on perishable foods and its benefits.

Highly perishable items such as meat and fish, dairy products, as well as fruits and vegetables, are some of the potential applications for these coatings. The edible coating surrounding the meat product prevents shrinkage, inhibits microbiological development, and prevents meat discoloration and unpleasant oxidative flavors (Figure 8). A variety of edible biopolymers are utilized to cover meat products [274,275]. Spraying and dipping the meat product in the coating-forming fluid produces the edible coating. When active ingredients are added to polysaccharides, they form active packaging that interacts with meat products and the environment, extending the shelf life of the meat product. Because alginate films are translucent, the consumer can see the colors and quality of the meat. In comparison to putting beautiful food-related artwork on the package, it is also an important component since buyers can view the product they will buy. According to studies, packaging transparency has an impact on customer purchasing decisions and bulk purchases [276]. Seafood has a short shelf life since it is very perishable. Because of the surrounding environment, seafood becomes contaminated during transit and storage, resulting in foodborne diseases and a change in the quality and nutritional content of the items. It also decreases seafood acceptability among consumers [134]. Fresh rainbow trout fish with a gelatin-based coating enhanced with cinnamon as an active antimicrobial agent is less likely to develop bacteria and thereby has a longer shelf life [277].

Butter is a high-fat dairy product with a short shelf life due to lipid oxidation. When kept at 2–5 °C, a corn-starch-based edible covering incorporating ginger oil inhibits lipid oxidation [278]. The addition of extract from *Satureja intermedia* to an apricot (*Prunus armeniaca*) gum coating has an antifungal effect against *Penicillium citrinum*, *Fusarium oxysporum*, *Aspergillus flavus*, and *Alternaria alternate*. Furthermore, the application of these active components in actual food has been investigated. During 60 days of storage, this coating successfully reduced the degree of microbial contamination caused by fungus on wild almond kernels [279].

Fruits and vegetables are, by far, the most popular coated products. This is due to their perishable nature, which has a significant influence on storage quality. As a result, coating technology is a viable strategy for extending the acceptability of fruits and vegetables among consumers as well as their shelf life (Figure 8). Aloe vera gel (AVG) is a polysaccharide-based natural biopolymer produced from aloe vera. For water and oxygen transmission, this gel works as a semipermeable membrane. It reduces the rate of respiration in fruits and vegetables, keeping the weight of the food product constant. It possesses antimicrobial and antioxidant qualities, extending the shelf life of fruits and vegetables. When this coating is applied to fruits or vegetables, it depletes the oxygen required for their metabolism. As a result, ripening and ageing are postponed, and the fruit or vegetable has a longer shelf life, even if the human consumes the coating. When AVG is applied to freshly cut papaya stored at 28 °C, it minimizes weight loss and keeps the papaya firm [280].

## 8. Conclusions and Future Perspective

Increasing environmental concerns have prompted research into the use of biodegradable packaging materials to replace traditional ones. In 2019, the global plastics output was estimated to be around 368 million tonnes. Plastic demand is on the rise due to the availability of takeaway and quick delivery services. However, poor recycling has mirrored the rise in plastic manufacturing, as seen in the European Union’s production of 61.8 million tonnes of plastic in 2018, compared to the recycling of just 9.4 million tonnes (https://www.plasticseurope.org/en/resources/market-data, accessed on 1 August 2022). It is important to ensure that the technology utilized to make edible films and coatings can be scaled up to meet such huge demands. With present findings, procedures such as solvent casting cannot be applied on a wide scale, and so more study is needed in this field. Furthermore, the raw materials used should be easily available, and therefore an emphasis should be placed on the use of industrial by-products as raw materials to contribute to long-term development. In this review paper, we looked at diverse biopolymer sources, active agents, and by-products that can be used to make films and coatings. All of these materials are food-grade and have been regarded as GRAS by the US-FDA. The properties of these materials, such as their moisture barrier, gas barrier, and mechanical and tensile strength, have contributed towards the development of films with enhanced attributes which may extend the shelf life of food products. These coatings can be applied as a single layer or in multiple layers, and they can be made from a variety of materials, improving the overall qualities of coatings and films. These films and coatings are applied to food products in a variety of ways: solvent casting and extrusion are employed for film production, and spraying, dipping, panning, and other processes are used for coating formation. These are directly applied to food goods and can be consumed with them. These operate as physical, chemical, and biological barriers, extending the shelf life of highly perishable foods such as meat, fruits, and vegetables. However, it is necessary to concentrate on the practical consequences of scaling up technologies utilized in the development of films and coatings in order to replace conventional technologies by lowering production costs and making them affordable. To attain more benefits of biopolymer-based films and coatings in food packaging applications, cost is a key factor that needs to be reduced for the packing of everyday products. According to recent reports, the prices of biopolymer-based films are in the range of USD 3–3.5/kg [281]. Based on a report of Jining Mingsheng New Material Co., Ltd., China, the approximate total cost for making bioplastic-based film is USD 3.6/kg [282].

## Figures and Tables

**Table 1:** Food packaging application from thermoplastics [9].

Thermoplastic Material	Abbreviation	Packaging Application	Pros and Cons
Polyethylene terephthalate	PET	Water/juice/soft drink bottles, food jars, microwavable containers, plastic films	Good mechanical strength and barrier properties, but low heat resistance and susceptible to oxidation
Polypropylene	PP	Drinking bottles for milk, food containers	Good chemical and moisture barrier, difficult to recycle
Polyvinyl chloride	PVC	Plastic bags, frozen foods, stretch films, container lids	Flexible, cost effective, difficult to recycle, but low heat resistance as adipates in PVC leach into food
Polystyrene	PS	Take-away clamshells, meat trays, bottle caps, straws	Easily recyclable, hard and brittle, but poor chemical resistance, can leach out into food when food is stored
Low-density polyethylene	LDP	Disposable cups, plates, and spoons; bread bags	Good chemical resistance, relatively permeable to oxygen but poor odor barrier
High-density polyethylene	HDP	Custom packaging, grocery bags, water/milk/juice containers, cereal and snack liners	Good moisture barrier, but poor gas barrier and low heat resistance

**Table 3:** Outline comparing dry and wet methods of film formation [187].

Sl. No.	Type of Method	Film Formation	Advantage	Disadvantage	Application
1.	Solvent casting method (Wet)	Biomaterial is dissolved in suitable solvent (e.g., ethanol, hexane) then cast into a mold followed by drying.	Low cost, ease of operation, fewer defects in film (homogeneous packaging), good optical purity, transparency, excellent flatness, and isotropic orientation.	Limitation of shapes, entrapment of toxic solvent in polymer, long drying period.	Apples, strawberries, quail eggs, cheese slices, etc.
2.	Extrusion method (Dry)	Biomaterial is mixed with solvent and then feed with the help of compressed air; it is then kneaded and heated in the machine, followed by the formation of the finished film.	Short duration of heating, low energy consumption, good mechanical, and optical properties, cheap and highly efficient, no use of solvent.	Can process materials that can tolerate high temperatures; high investment, and maintenance cost.	Mangos, soyabean oil, sausage

## Data Availability

Not applicable.
